# Can self-assessment and augmented feedback improve performance and learning retention in manual therapy: results from an experimental study

**DOI:** 10.1186/s12998-023-00505-0

**Published:** 2023-09-12

**Authors:** Mégane Pasquier, Sahel Memari, Arnaud Lardon, Martin Descarreaux

**Affiliations:** 1 Research Department, Institut Franco-Européen de Chiropraxie, 24 Boulevard Paul Vaillant Couturier, 94200 Ivry-Sur-Seine, France; 2 Department of Human Kinetics, Université du Québec À Trois-Rivières, 3351 Bd Des Forges, Trois-Rivières, QC G8Z 4M3 Canada

**Keywords:** Motor skills, Motor learning, Spinal manipulation, Feedback

## Abstract

**Background:**

The purpose of this study was to investigate how feedback and self-assessment strategies affect performance and retention of manual skills in a group of chiropractic students.

**Methods:**

Seventy-five students participated in two spinal manipulation (SM) learning sessions using a force-sensing table. They were recruited between May and November 2022 during HVLA technical courses. Students were randomly assigned into three different groups: participants in group 1 received visual feedback, those in group 2 received visual feedback after self-assessment, and participants in group 3 (C) received no feedback. During the first session, participants started with one block of 3 familiarization trials, followed by two blocks of 6 SM HVLA (high velocity low amplitude) posterior-to-anterior thoracic SM trials, with 3 trials performed with a target force of 450 N and 3 others at 800 N. They received feedback according to their group during the first block, but no feedback was provided during the second block. All participants were invited to participate in a second session for the retention test and to perform a new set SM without any form of feedback.

**Results:**

Results showed that visual feedback and visual feedback in addition to self-assessment did not improve short-term SM performance, nor did it improve performance at the one-week retention test. The group that received visual feedback and submitted to self-assessment increased the difference between the target force and the peak force applied, which can be considered a decrease in performance.

**Conclusion:**

No learning effects between the three groups of students exposed to different feedback and self-assessment learning strategies were highlighted in the present study. However, future research on innovative motor learning strategies could explore the role of external focus of attention, self-motivation and autonomy in SM performance training.

## Introduction

Over the past few decades, numerous studies have focused on teaching, learning and motor control methods related to manual therapy skills such as spinal manipulation and mobilization; hereafter referred to as spinal manipulation (SM) [1, 2]. Spinal manipulations can be compared to movements often performed in sports; they share similarities with ballistic skills which involve forceful movements that reach peak acceleration within milliseconds of their initiation. Some of the spinal manipulation skills require complex movements that are initiated proximally using trunk muscles followed by energy that is transferred from one segment to another one, to the upper limbs. Therefore, learning spinal manipulation can represent a serious challenge to some students.

According to Schmidt, learning is defined, as “a set of operations associated with practice or experience, which lead to relatively permanent changes in competence for the performance of motor skills” [3]. Learning can be subdivided into three phases according to Fitts [4]: I) the cognitive or verbal motor phase II) the associative or motor phase and III) the so-called autonomous phase. In order to acquire these skills, variability, constant or random practice, feedback and repetition are essential elements of learning. In 2016, a review listed the various strategies that improve the performance of manual therapists [2]. The review highlighted the positive effect of feedback training in the context of manual therapy training. More recent evidence also supports the use of objective feedback for SM learning [5, 6]. This feedback is most often delivered by experienced instructors, videos or instrumented devices providing information about the force–time profile of the manual therapy task being performed [2]. However, despite recent evidence regarding motor learning of manual therapy skills, which suggests the importance of feedback in short-term performance improvement and skill retention, there is very little evidence regarding the timing, relevance, context and organization of reinforced feedback training for manual therapy skills [7].

Yet, several factors can improve motor learning and lead to expertise. Both motivation to learn a new motor task and autonomy in the form of self-controlled practice are associated with improved learning and retention [8]. Chiviacowsky (2002) [9] showed, in a study investigating why self-controlled feedback is effective, that learners under self-controlled conditions can adequately estimate their errors and discriminate between better or worst performance.

In an effort to integrate the social, cognitive and affective components of motor learning, Wulf and Lewthwaite proposed, in 2016, the “Optimizing Performance Through Intrinsic Motivation and Attention for Learning” (OPTIMAL) Theory [10]. One of the premises of the theory is that active participation in the learning process appears to optimize it. For example, Hattie and Temperley [11] argue that self-assessment can be used by learners as a self-regulatory skill that can stimulate learning. In the field of education, self-appraisal is considered one of the of self-assessment components, and is defined as the learner’s ability to evaluate their abilities, knowledge, and cognitive strategies through a variety of self-monitoring processes [12].

Although the benefits of feedback training and self-monitored practice have been demonstrated in various learning contexts, Wulf (2007) [8] notes that future healthcare providers learning manual skills often assume a passive role in their learning, and that, even with the inclusion of augmented feedback, providing self-control over practice to learners does not seem to be a frequent teaching approach. The objective of the present study was therefore to investigate how augmented feedback and self-assessment strategies affect performance and retention of manual skills in a group of manual therapy (chiropractic) students. It was hypothesized that students exposed to feedback and self-assessment learning strategies would perform better in the manual skill retention test.

## Methods

### Study design

This one-week prospective study was approved by the Institut Franco-Européen de Chiropraxie ethics committee (CER-15–215-07.07). Each participant provided a written informed consent prior to the first experimental session of the study. Data were collected between October 2022 and January 2023.

### Participants

A total of 75 chiropractic students participated in this study. The students were recruited during their third, fourth or fifth study year (5-year study), on two different campuses between May and November 2022 during HVLA technical courses. The participants received explanations and instructions regarding the experimental protocol prior to the first experimental session, and they were told they would be exposed to one of the different SM teaching strategies.

### Experimental procedure

Participants were randomly assigned, using www.randomization.com, into three different groups. Participants in group 1 received visual feedback (ViF), whereas participants in group 2 were asked to self-assess their performance and received visual feedback after self-assessment (S-A). Participants in group 3 did not receive any form of feedback and were considered the control group (C). The experimental protocol included two sessions performed seven days apart.

For all participants, the *first session* began with a block of 3 familiarization trials, followed by two blocks of 6 SM trials (Block 1 session 1). The SM familiarization trials were performed by participants on a manikin (H.A.M. series; Canadian Memorial Chiropractic College, Toronto, ON) without any target force and using the HVLA posterior-to-anterior thoracic SM procedure of their choice. The second block (Block 2 session 1) included 6 HVLA posterior-to-anterior thoracic SM trials, with 3 trials performed with a target force of 450 N and 3 other trials with a target force of 800 N. The trial sequences were randomized per block (using the www.randomization.com website) and the trial target force was provided to the participant before each trial by an experimental instructor. To standardize time per trial in all groups, each trial lasted 30 s, including feedback and self-assessment when applicable.

During the second session of trial (Block 3 session 2), participants in the control group did not receive any feedback, while the ViF group received visual feedback for peak force value and SM force–time profile after each trial. Participants in the S-A group were asked to estimate their own applied total peak force in newtons just after the execution of each SM trial, and to indicate if this value was on target (correct: ± 10%), too low (> 10%) or too high (> 10%). After the estimation, participants in the S-A group also received visual feedback for peak force value and SM force–time profile. The first experimental session included a third block for all participants. This third block of 6 trials was conducted to assess SM performance following the intervention or control session of three groups; it included 6 trials (randomized target force of 450 N or 800 N) without any feedback regarding the participant’s performance.

### Retention test

**Fig. 1:**
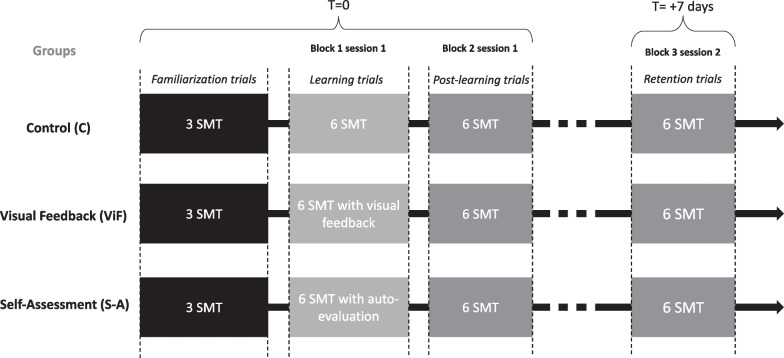
Experimental procedure for each group after randomization

All participants were invited to participate in a *second experimentation session* of 7 days after their *first one*. The *second session* of the experimental protocol was used to assess learning retention; it included, for all participants, one familiarization trial, followed by a block of 6 SM trials (randomized target of 450 N or 800 N). During this block, participants did not receive any feedback concerning their performance. Figure 1 illustrates the two experimental sessions and the overall design of the study.

### Experimental device

All SM trials were performed on a manikin made of a plastic spine and high-density foam padding that permitted anteroposterior compression of the thorax and for which skeletal landmarks were palpable through the foam. This instrument has been used in previous SM learning studies [13, 14]. A Force Sensing Table Technology® system (FSTT®, Canadian Memorial Chiropractic College, Toronto, ON) composed of a Leander 900 Z Series chiropractic table (Leander Health Technologies Corporation, Lawrence, KS) and an embedded force plate (AMTI, Watertown, MA) was used to measure the input forces. All transmitted forces were computed in a XYZ coordinate system using the FSTT® software (Canadian Memorial Chiropractic College, Toronto, ON).

### Data acquisition

The vertical Force–time signals (Fz) applied on the table during HVLA manipulations were obtained for each trial, and relevant SM biomechanical parameters for each trial were analyzed. The following parameters were used for subsequent analyses (i) Preload force (N): the amount of force applied prior to the thrust, (ii) Peak force (N): the maximal force applied during the thrust, (iii) time to peak force (ms): the time needed to reach peak force, iv) Rate of force application (N/s): the ratio between peak force and time to peak force.

Using the target force and the peak force applied, the following variables were also computed: (i) Constant Error (CE): the positive or negative difference between the peak force reached and the peak force targeted; (ii) Absolute Error (AE): the absolute deviation from the targeted force, regardless of direction; and (iii) Variable Error (VE): the participants’ consistency, which was defined as the absolute value obtained by subtracting the peak force reached during each trial from the participant’s mean peak force.

### Statistical analysis

Baseline group characteristics, including SM force–time parameters for the first 450 N trial, were calculated. Age, gender, year of study, as well as peak force, time to peak force and rate of force production were then compared between the 3 groups. Normal distribution was established using the Shapiro-Wilks test and visual inspection. A mixed-model analysis of variance (ANOVA) was performed to assess Group, Time and Group x Time interaction effects for all SM variables, and a Greenhouse-Houser adjustment was used whenever the assumptions of sphericity were not met. Post-hoc analyses were performed using Tukey’s test.

The percentage of accurate estimations (± 10% of the reached peak force), as well as the mean absolute difference between the estimation and the reached peak force were calculated for each participant of the S-A group. The significance level for all analyses was set to p < 0.05. Statistical computations were performed using IBM SPSS Statistics version 28.0.0.

## Results

### Participants’ characteristics

A total of 75 students participated in the study which included 39 women and 36 men, with a mean age of 22.7(± 2.6) years old. Twenty-three, 28 and 24 participants were respectively included in ViF group, S-A Group and the C Group. For the first 450 N trial, mean group forces were 508.1 (± 103.7) N, 496.2 (± 90) N, 511 (± 106) N for ViF, SA Group and the C Group. Participants’ characteristics for each group are presented in Table 1. Group comparison analyses showed that age, gender, year of study as well as SM force–time parameters for the first 450 N trial were similar between the 3 groups.

**Table 1:** Participants Characteristics (Mean ± SD)

	Total sample [n = 75]	Visual Feedback group [n = 23]	Self-Assessment group [n = 28]	Control group [n = 24]	*P* value*
Age (y)	22.7 ± 2.6	23.1 ± 3.4	22.6 ± 2.1	22.5 ± 2.4	0.7
Women (n (%))	39 (52%)	12 (52%)	18 (64%)	9 (37%)	0.15
Men (n (%))	36 (48%)	11 (48%)	10 (36%)	15 (63%)	
Year of study					
3 (n)	38	13	16	9	0.53
4 (n)	25	7	7	11	
5 (n)	12	3	5	4	
1st (450N) trial peak force (N)	504.6 ± 98.4	508.1 ± 103.7	496.2 ± 90.0	511.0 ± 106.0	0.85
1st trial time to peak force (ms)	132.4 ± 16.3	136.0 ± 15.6	132.0 ± 16.8	129.5 ± 16.2	0.38
1st trial rate of force application (N/S)	2651 ± 741	2616 ± 731	2728 ± 772	2596 ± 737	0.79

*P values indicate the results of the one-way ANOVA for age and SM force–time profile variables and Chi-square test for gender and year of study

### SM biomechanical parameters

For the 450N target, all groups reached and exceeded the 450N (for all blocks of trials) whereas none of the groups reached the 800N target for any of the blocks. Corresponding SM biomechanical parameters are presented in Tables 2 and 3.

**Table 2:** Peak force means (± SD for each group at Session 1 and 2 for target 450 N

Peak force	Block 1 Session 1	Block 2 Session 1	Block 3 Session 2
Group 1 (ViF)	487.5 ± 16.8	488.1 ± 18.9	471.3 ± 17.1
Group 2 (S-A)	472.8 ± 15.4	485.9 ± 17.3	535.8 ± 15.6
Group 3 (C)	493.4 ± 16.0	449.9 ± 18.0	464.4 ± 16.3

Time to peak	Block 1 Session 1	Block 2 Session 1	Block 3 Session 2
Group 1 (ViF)	140.2 ± 3.2	131.5 ± 3.4	133.2 ± 4.9
Group 2 (S-A)	130.0 ± 2.9	135.9 ± 3.1	137.6 ± 4.5
Group 3 (C)	131.3 ± 3.0	132.5 ± 3.3	139.0 ± 4.7

Rate of force	Block 1 Session 1	Block 2 Session 1	Block 3 Session 2
Group 1 (ViF)	2404.0 ± 121.0	2453.3 ± 131.0	2360.8 ± 136.4
Group 2 (S-A)	2483.3 ± 111.0	2416.6 ± 120.1	2659.3 ± 125.0
Group 3 (C)	2395.5 ± 130.3	2291.4 ± 125.2	2218.5 ± 130.3

**Table 3:** Peak force means ($\pm \mathrm{SD})$ for each group at Session 1 and 2 for target 800 N

Peak Force	Block 1 Session 1	Block 2 Session 1	Block 3 Session 2
Group 1 (ViF)	614.8 ± 26.7	625.3 ± 28.2	637.8 ± 27.3
Group 2 (S-A)	618.5 ± 24.0	591.8 ± 25.4	625.9 ± 24.6
Group 3 (C)	670.5 ± 26.1	672.7 ± 27.6	682.2 ± 26.7

Time to peak	Block 1 Session 1	Block 2 Session 1	Block 3 Session 2
Group 1 (ViF)	138.8 ± 3.7	140.4 ± 3.3	142.9 ± 3.5
Group 2 (S-A)	140.1 ± 3.4	135.3 ± 3.0	136.2 ± 3.1
Group 3 (C)	132.4 ± 3.7	133.6 ± 3.2	134.2 ± 3.4

Rate of force	Block 1 Session 1	Block 2 Session 1	Block 3 Session 2
Group 1 (ViF)	3485.5 ± 224.4	3488.3 ± 243.6	3388.1 ± 211.1
Group 2 (S-A)	3535.2 ± 201.6	3421.1 ± 219.0	3573.6 ± 189.6
Group 3 (C)	3834.1 ± 219.2	3855.9 ± 238.1	3798.5 ± 206.1

**Fig. 2:**
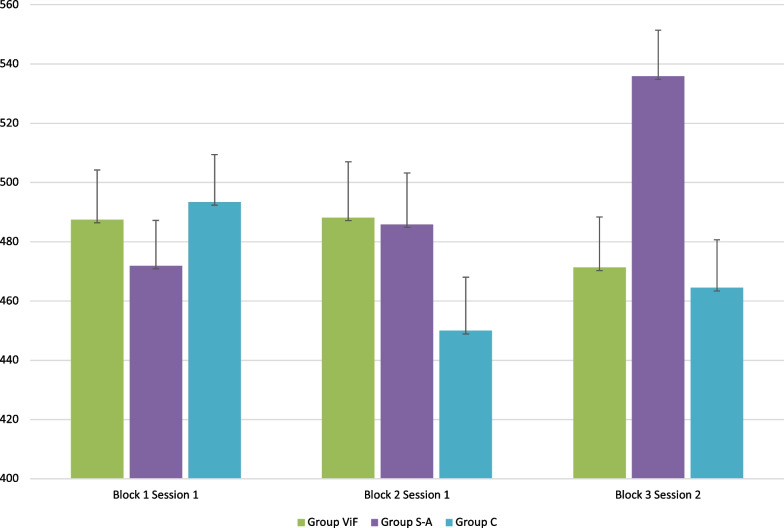
Mean peak force per group at each block (mean values and standard deviation)

Mixed-model analyses showed, for the 450 N target a significant Group X Time interaction for the peak force values (*p* = 0,015, ηp^2^ = 0.09). Post-hoc analyses showed that participants in the S-A group significantly increased peak force values during the retention test (Block 1: 472.8N (± 15.4); Block 2: 485.9N (± 17.3); Block 3: 535.8N (± 15.6)) compared to the other two groups. Figure 2 shows changes in peak force throughout the trial blocks for the 3 groups. No Group, Time or Group x Time interaction effects were found for time to peak and rate of force application variables at 450 N target. For the 800 N target, no significant Group, Time or Group x Time interactions were observed for any of the biomechanical parameters (ps > 0.05).

### Constant error, absolute error and variable error

Time effect was observed for absolute error for the 450 N target (p = 0.013) and the 800 N target for all groups (p = 0.018). Indeed, post-hoc analyses showed that the S-A group was the only one showing a significant increase in absolute error at block 3 compared to block 1 and 2 (Block 1:54.6 N (± 9.7); Block 2:68.5 N (± 10.7); Block 3:128.5 N (± 17.5)). Time effect was also observed for variable error for the 450 N target for all groups (*P* < 0.0001). A Group x Time interaction for absolute error for the 450 N target was observed (p = 0.026). All other analyses showed no significant effect $(ps>0.05)$.

### Peak force estimation

The percentage of accurate estimations in the S-A group was 50% (1.5 trials accurately estimated over 3) for the 450 N trials and 33% (1 trials accurately estimated over 3) for the 800 N trials. The mean absolute difference between the estimation and the reached peak force was 44.7 (± 37.8) N for the 450 N trials and 60.9 (± 41.5) N for the 800 N trials.

## Discussion

The overall goal of the study was to assess the impact of augmented feedback and self-assessment strategies on the performance and retention of SM skills. Skills were assessed through a series of SM force–time profile variables, but also using bias, accuracy and consistency measurements. Results showed that visual feedback regarding SMT force–time profile performance (ViF group) alone or combined with self-estimation of SM performance (S-A group) did not improve short-term SM performance, nor did it improve performance at the one-week retention test, although previous studies have shown that augmented feedback can be useful when learning spinal manipulation techniques. A study by Pasquier et al. (2017) investigated the effect of augmented feedback on SM biomechanical parameters using global performance feedback provided verbally and reinforced, when necessary, by specific quantified feedback related to SM force–time profile characteristics [7]. In this previous study, feedback led to significant improvement in the rate of force applications, preload force and the drop in preload force, but failed to identify learning effects for peak force and time-to-peak force, which is consistent with the current results. Interestingly, the S-A group increased its peak force during the retention block (block 3), but increased the difference between the target force and the peak force applied, which can be considered a performance decrease. Although previous study have shown that small regimen of augmented feedback can improve SM learning [7, 15, 16], the limited number of practice trials with augmented feedback for each force target may have been insufficient to trigger learning effects. Moreover, according to the guidance hypothesis, augmented feedback can lead to error corrections and subsequent learning but providing inadequate feedback (e.g. timing or quantity) can have a detrimental effect on learning. Schmidt (1991)[17] suggested that providing frequent (too frequent) augmented feedback to the learner can lead to too many corrections during practice (maladaptive short-term corrections), which can be responsible for an inability to recognize and produce stable behavior during retention tests. It is therefore important for learners to gradually reduce their reliance on external feedback as they become more skilled at a particular motor task, in order to develop greater independence and self-regulation in their movements. Finally, self-estimating a performance combined with systematic augmented feedback may have created an information overload also known to be detrimental to motor learning in novices [18].

This is the first study to investigate the effect of self-estimation strategy on SM performance and learning. Although a review by Wulf et al. (2010) [19] suggests that directing attention to the effect of the motor task on the environment (external focus) generally results in more effective performance and learning, the present study failed to identify significant SM learning or retention effects. The lack of significant improvement following visual feedback and self-estimation strategies may be due to the relative inexperience of the participants with the task and the limited number of trials with augmented feedback. Indeed, it has been argued that when learning a new and relatively complex motor skill, directing attentional focus to a specific aspect of the task (in this case, the target force) may be detrimental to the overall performance [20]. Directing participants to specific components of the tasks (even when it is a critical component) may hinder the processing of important information required for learning, and even interfere with the cognitive demands related to the task [20]. An additional potential explanation for the lack of learning effects is a possible physical limitation that restricted the desired movement outcome. A few participants were unable to produce sufficient forces to reach the target forces, and were therefore unable to use the proposed learning strategies. For instance, the 800 N was deemed to be a challenging condition for participants but may have been “too challenging” for novices’ performer. According to the challenge point theory, performance is expected to decline rapidly as task difficulty increases [21].

Regarding force target reaching and estimations, Starmer et al. (2016) [14] showed that first-year chiropractic students were, on average, 11% off-target (400 N target) and 21% off-target (600 N target) in a similar SM task. These results combined with those of the present study suggest that force control and transferability, as well as error detection skills, are most likely mastered in later stages of SM learning, as previously suggested [22].

### Strength and limitations

This study was the first to explore the potential benefits of learning strategies promoting external focus of attention, as well learners’ autonomy and self-regulation. Although the results failed to identify significant short-term performance and learning effects, it provided a template to implement similar studies with extended learning and assessment periods. Allowing students to design, within a given learning template, their practice schedule and the nature and timing of feedback, may still be beneficial if properly integrated in the teaching curriculum.

One limitation of the study was the restricted number of trials per condition, which may have affected the overall precision and variability of the data, and potentially contributed to the lack of differences between the groups. Blocks of 3 trials were chosen to encourage participation within the teaching environment, and to limit the possible sequence effects due to online learning (change in mean performance due to trial repetition). Finally, short-term learning studies have been used to explore the potential of learning strategies that could be implemented over a longer period of time, and their results should be interpreted with caution. The effects of long-term feedback and force target estimation training strategies remains to be investigated.

## Conclusion

Overall, this study was the first to explore the effect of feedback and self-estimation strategies on SM performance and learning. Although the chosen design and feedback and self-assessment learning strategies did not lead to improved SM skills at the retention test, it highlighted the need for precise strategies targeted at improving specific components of SM skills. Future studies should explore the potential of strategies involving external focus of attention, self-motivation and autonomy to improve SM performance.

## Data Availability

All data generated or analyzed during this study are included in this published article Data can be made available upon request.

## Abbreviations

SM: Spinal manipulation
C: Control group
S-A: Self-assessment group
ViF: Visual feedback
HVLA: High velocity low amplitude
CE: Constant error
VE: Variable error
AE: Absolute error
SD: Standard deviation
